# Burden of cardiovascular disease from 1990 to 2017 in Henan Province, China

**DOI:** 10.1080/16549716.2021.1959708

**Published:** 2021-08-23

**Authors:** Yan-fang Zhao, Tai Zhang, Zhuo-qun Wang, Xiao-rong Chen, Chun-xiao Wang, Jin-lei Qi, Jing Yang, Jing Wu, Mai-geng Zhou

**Affiliations:** a Division of Science, Education & International Cooperation, National Center for Chronic and Non-communicable Disease Control and Prevention, China CDC, Beijing, People’s Republic of China; b Epidemiology and Biostatistics Unit, School of Public Health, Dali University, Dali, People’s Republic of China; c Division of Cardiovascular Disease Control and Prevention, National Center for Chronic and Non-communicable Disease Control and Prevention, China CDC, Beijing, People’s Republic of China; d Division of Oral Health, National Center for Chronic and Non-communicable Disease Control and Prevention, China CDC, Beijing, People’s Republic of China; e Division of Vital Registry and Mortality Surveillance, National Center for Chronic and Non-communicable Disease Control and Prevention, China CDC, Beijing, People’s Republic of China; f National Center for Chronic and Non-communicable Disease Control and Prevention, China CDC, Beijing, People’s Republic of China

**Keywords:** Cardiovascular diseases, burden of disease, disability-adjusted life years, risk factors

## Abstract

**Background:**

Cardiovascular diseases (CVDs) are the leading causes of death in China. Little is known about the CVD burden and risk factors in Henan Province, China.

**Objective:**

To analyze the CVD burden and main risk factors between 1990 and 2017 in the Henan Province in China.

**Methods:**

The methodological framework and analytical strategies adopted in the Global Burden of Disease Study 2017 were used.

**Results:**

(1) Age-standardized mortality rate attributed to CVDs increased from 355.0 per 100,000 persons in 1990 to 364.1 per 100,000 persons in 2017 in Henan. (2) Age-standardized disability adjusted life years (DALYs) rate fell by 3.9% from 1990 to 2017. However, the number of DALYs attributed to CVDs increased by 75.9% from 4.2 million person-years in 1990 to 7.3 million person-years in 2017. (3) The age-standardized years lived with disability (YLDs) rate increased by 27.5% from 1990 to 2017. However, years of life lost (YLLs) rate decreased by 6.7% from 1990 to 2017. The contribution of YLLs to the DALYs decreased from 91.4% in 1990 to 89.2% in 2017. (4) Stroke (52.3%) and ischemic heart diseases (38.8%) accounted for 91.1% of total CVDs DALYs among adults in 2017. (5) Dietary factors such as high intake of sodium, alcohol use and low intake of fruits, high systolic blood pressure, and tobacco use were the top risk factors for CVDs, and the estimated population attributable fraction in 2017 was 69.4%, 56.7% and 25.2%, respectively.

**Conclusions:**

The absolute burden of CVDs in Henan is still high, although age-standardized DALYs declined between 1990 and 2017. The prevention and control of stroke and ischemic heart diseases should focus on a few modifiable risk factors which mainly contributed to the burden of CVDs, such as dietary factors, high systolic blood pressure, and tobacco use.

## Introduction

Cardiovascular diseases (CVDs) and risk factors are the leading causes of deaths and disease burden worldwide. In 2016, a total of 17.9 million people died from CVDs globally, representing 31% of total global deaths [1]. CVDs are also the major causes of premature deaths under 70, contributing to 37% of total global premature deaths due to noncommunicable diseases [1]. In China, the number of prevalent CVD cases reached nearly 94 million in 2016, resulting in about 3.97 million deaths and 78.11 million disability-adjusted life years (DALYs), which were more than one in three of the global CVD burden [2]. In 2016, 20.02 million patients with CVDs were discharged from hospitals in China, and the total hospitalization expenditures exceeded 100 billion Chinese Yuan (CNY, 1 CNY = 0.15 USD), accounting for 2.26% of the national health expenditures [3]. Among the discharged patients with CVDs, ischemic heart diseases (7,382,400) and cerebral infarction (6,403,000) were the leading causes of hospitalizations, accounting for 36.87% and 31.98% of all admissions, respectively [3].The average length of stay of patients with hemorrhagic stroke in 2017 was 14.5 days, with an average medical fee of 18,524.6 CNY, while that of patients with ischemic stroke was 10.7 days, with an average medical fee of 9607.0 CNY [4]. Therefore, CVDs brought a huge burden on disability, death, and the economy, heavily impacting individuals, families, and society.

Due to the differences in social and economic development, geographical environment, dietary habits, health resources, and health services in different provinces of China, there are significant provincial differences in the disease burden. Although previous studies have reported a varied epidemiological transition at the national level of China [2] there are few systematic analyses of the CVD burden at the provincial level. The characteristics of disease burden and risk factors of CVDs at provincial level in China are important for understanding the local CVDs epidemiological situation and formulating prevention and control strategies.

Henan Province is located in mid-eastern China, with a population of more than 100 million, accounting for 7.8% of the China’s total population. The economy of Henan Province is in the rising stage, with obvious characteristics of rapid urbanization, population aging, and lifestyle transformation. A study has shown that heart disease and CVDs accounted for 5.32 years and 4.71 years lost in Henan Province residents’ life expectancy in 2014. CVDs made the most significant impact on the life expectancy of Henan residents [5]. However, there are few reports on the CVDs burden and its risk factors in Henan, which are urgently needed for determining targeted interventions to ensure sustainable development of local economy. This study aimed to analyze the disease burden and major CVD risk factors between 1990 and 2017 in Henan Province.

## Methods and data sources

### Overview

The full detailed methods of the Global Burden of Disease Study 2017 have been published in previous reports [6–11]. Specifically, a wide range of updated and standardized analytical procedures were used for years of life lost (YLLs) and years lived with disability (YLDs) estimation [6,7,10]. Some of the results in Henan were directly extracted from the Chinese estimates by province in the Global Burden of Disease (GBD) Study 2017.

### Data sources

The age-specific and sex-specific population data were extracted from censuses, annuals and sample surveys conducted by the National Bureau of Statistics. The population sizes of all 34 provinces were estimated with a Bayesian hierarchical model to ensure maximum internal consistency [9]. The data on death causes were from the Chinese estimates in GBD 2017, which used data from the Disease Surveillance Point System and the Maternal and Child Surveillance System, the China Cancer Registry, and the cause-of-death reporting system of Chinese Center for Disease Control and Prevention [9].The data on prevalence and incidence of non-fatal outcomes were derived primarily from national surveys, such as the Fifth National Health Service Survey and the Chronic Disease and Risk Factor Surveillance, cancer registries, and hospital inpatient data and published studies [9].

### Specific methods

#### Disease burden estimation

The prevalence of each cause and the distribution by severity were primarily estimated with the Bayesian meta-regression method DisMod-MR 2.1 [7,11]. Regression models were used to adjust data that did not follow the standard definition for each cause in the direction of case definition–based data [12]. The all-cause and cause-specific death rates were estimated by the Cause of Death Ensemble model [11]. YLDs, YLLs, and DALYs were estimated with the methods in the published literature [7,10,11,13–17]. Briefly, YLDs for a specific cause was calculated by multiplying its prevalence with the corresponding disability weight, which had been estimated in several previous worldwide surveys and adjusted for comorbidity. YLLs was calculated by summing up the remaining life expectancy of people dying in each age group. DALYs equaled to the sum of YLLs and YLDs by any corresponding sub-population of a specific cause [7,10,11,13–17]. Age-standardized mortality rates, YLLs rates, YLDs rates, and DALYs rates were computed based on the world standard population reference developed for GBD study, which was a time-in-variant standard [11,18].

### Population Attributable Fractions (PAFs)

The disease burden attributable to different risk factors was estimated based on the comparative risk assessment (CRA) framework [6,19]. Briefly, the principle of CRA framework was that when the exposure level of other independent risk factors was constant, the exposure distribution of a certain risk factor in a specific population was compared with the theoretical-minimum-risk-exposure distribution to obtain the proportion of disease burden attributed to this risk factor, which was PAFs [6,19]. Then the DALYs of a specific disease was multiplied by the PAFs of the risk factor to get the disease burden attributed to this risk factor. Therefore, the PAFs represented the proportion of outcomes that would be reduced in any given year if exposure to a risk factor in the past had been reduced to the counterfactual level of theoretical-minimum-risk-exposure [6].

The estimation methods, exposure definition, theoretical-minimum-risk-exposure level, and PAFs of the factors referred to previous studies [6,19,20].

PAFs formula for the risk factors as continuous variables was shown below [6]:

$$PAFs=\frac{\int_{x=0}^{m}RR\left(x\right)P_{1}\left(x\right)dx-\int_{x=0}^{m}RR\left(x\right)P_{2}\left(x\right)dx}{\int_{x=0}^{m}RR\left(x\right)P_{1}\left(x\right)dx}$$

Among them, RR(x) was the relative risk when the exposure level was x. P1(x) was the current exposure distribution of a population, P2(x) was the theoretical-minimum-risk-exposure distribution, and m was the highest exposure level.

PAFs formula for the risk factors as categorical variables was shown below [6]:

$$PAFs=\frac{\sum_{i=1}^{n}P_{i}\left(RR_{i}-1\right)}{\sum_{i=1}^{n}P_{i}\left(RR_{i}-1\right)+1}$$

Among them, RR_i_ was the relative risk when the exposure level was i, P_i_ was the exposure rate of the population when the exposure level was i, and n was the number of exposure levels. When it was attributed to one or more risk factors simultaneously, the PAFs estimation assumed that each risk factor was independent and could be calculated by the PAFs of individual risk factor. The PAFs formula applied to the case of multiple risk factors [6]:

$PAFs=1-{\Pi^{R}}_{r=1}\left(1-PAF_{r}\right)$, Where r was the individual risk factor and R was the number of risk factors.

### Uncertainty Intervals (UIs)

The 95% uncertainty interval was reported for all the indicators of disease burden in the analysis. These uncertainty intervals were propagating uncertainty through the estimation chain using posterior simulation with 1000 draws, from which the lower and upper bounds of the UIs were derived based on the 2.5th and 97.5th percentiles [6,7,10,11]. The 95% uncertainty intervals were measured by the method described in the reference section [6,7,10,11].

## Results

### CVDs cases and prevalence

The age-standardized prevalence rate for CVDs in Henan Province increased by 11.6%, from 5668.4 per 100,000 (95% UI: 5461.8–5905.2) in 1990 to 6325.9 per 100,000 (95% UI: 6086.6–6578.4) in 2017, among which that of males increased by 13.3% from 5378.7 per 100,000 (95% UI: 5159.5–5613.9) to 6094.8 per 100,000 (95% UI: 5828.8–6370.1), and that of females increased by 10.0% from 5942.5 per 100,000 (95% UI: 5701.3–6190.1) to 6539.6 per 100,000 (95% UI: 6269.6–6808.7) (Table 1).

**Table 1.** Cases, prevalence, and percentage change between 1990 and 2017 for genders combined and separated for cardiovascular diseases in Henan

Groups	No. of cases per 100,000(95% UIs)			Age-Standardized Prevalence (95% UIs), per 100,000		
	1990	2017	Percentage Change (%)	1990	2017	Percentage Change (%)
Both genders						
Cardiovascular diseases	3923(3775–4085)	7546(7259–7869)	92.3	5668.4(5461.8–5905.2)	6325.9(6086.6–6578.4)	11.6
Rheumatic heart disease	672(636–708)	787(748–829)	17.2	758.0(718–798.1)	756(718.7–796.9)	−0.3
Ischemic heart disease	765(713–820)	1517(1415–1639)	98.4	1190.7(1110.1–1275.7)	1263.3(1174.2–1360.2)	6.1
Stroke	1150(1087–1215)	2693(2515–2884)	134.1	1641.6(1548.1–1736.2)	2211.9(2067–2363.5)	34.7
Hypertensive heart disease	171(143–201)	398(333–470)	133.0	285.7(239.3–339.2)	343.1(287.1–404.1)	20.1
Cardiomyopathy and myocarditis	10(8–13)	16(13–20)	61.3	14.1(11.3–16.9)	15.1(12.2–18.1)	7.4
Atrial fibrillation and flutter	220(188–254)	477(408–551)	116.7	379.6(324.3–437.3)	416(356.1–481)	9.6
Aortic aneurysm						
Peripheral artery disease	891(766–1032)	1783(1546–2052)	100.1	1436.7(1239.8–1655.6)	1453(1259.8–1659.8)	1.1
Endocarditis	2(2–3)	4(3–4)	58.9	3.1(2.7–3.5)	3.7(3.2–4.1)	18.9
Non-rheumatic valvular heart disease	89(83–96)	198(185–211)	121.6	142.8(134.3–152.1)	167.3(157.4–177.2)	17.2
Other cardiovascular and circulatory diseases	284(230–338)	541(435–662)	90.6	379.2(307.1–455.2)	461.9(377.2–552.4)	21.8
Males						
Cardiovascular diseases	1792(1719–1870)	3457(3309–3628)	92.9	5378.7(5159.5–5613.9)	6094.8(5828.8–6370.1)	13.3
Rheumatic heart disease	306(288–325)	338(319–358)	10.4	671.9(632.1–711)	661.2(623.7–700.1)	−1.6
Ischemic heart disease	356(329–384)	670(615–730)	88.2	1186.7(1099.9–1279.1)	1189.6(1095.4–1290.4)	0.2
Stroke	542(508–576)	1351(1250–1465)	149.4	1590.3(1481.1–1694.5)	2335.1(2160.5–2520.5)	46.8
Hypertensive heart disease	60(50–72)	142(116–170)	135.6	211.7(175.2–253)	257.8(212.2–307)	21.7
Cardiomyopathy and myocarditis	5(4–7)	8(6–10)	37.2	15.2(11.5–19.6)	14.2(10.7–17.9)	−6.5
Atrial fibrillation and flutter	96(82–111)	210(179–243)	117.9	375.7(319.3–432.8)	400.6(341.4–464.2)	6.6
Aortic aneurysm						
Peripheral artery disease	381(328–444)	765(656–878)	100.8	1323(1149.5–1528.2)	1330.6(1143.3–1525)	0.6
Endocarditis	39(36–43)	2(2–2)	55.4	3.4(3–3.8)	4(3.5–4.5)	17.5
Non-rheumatic valvular heart disease	139(113–167)	88(80–95)	123.6	134.8(124.3–145.5)	159.4(147.5–171.6)	18.2
Other cardiovascular and circulatory diseases	3457(3309–3628)	256(207–316)	84.8	378.2(307.1–455.4)	452.7(367.1–550.2)	19.7
Females						
Cardiovascular diseases	2131(2044–2217)	4089(3915–4266)	91.9	5942.5(5701.3–6190.1)	6539.6(6269.6–6808.7)	10.0
Rheumatic heart disease	365(344–388)	449(423–475)	22.9	847.4(798.8–899.5)	850(799.6–901.1)	0.3
Ischemic heart disease	409(380–439)	848(787–915)	107.4	1202.1(1118.6–1289.6)	1329.4(1233.1–1436.4)	10.6
Stroke	609(572–647)	1342(1245–1445)	120.5	1682.3(1581.7–1785.4)	2101.7(1953.8–2258.1)	24.9
Hypertensive heart disease	111(91–132)	256(215–303)	131.6	339.4(281.2–405.1)	412.4(346.6–488.2)	21.5
Cardiomyopathy and myocarditis	5(3–6)	9(7–11)	89.7	12.8(9.5–16.3)	16.3(12.2–20.7)	27.0
Atrial fibrillation and flutter	124(106–144)	267(228–310)	115.7	384.2(328.5–443.9)	430.7(367.8–500.2)	12.1
Aortic aneurysm						
Peripheral artery disease	510(437–591)	1018(882–1173)	99.5	1541.2(1322.6–1773.8)	1561(1355.3–1794.1)	1.3
Endocarditis	1(1–1)	2(2–2)	63.1	2.8(2.5–3.1)	3.4(3–3.8)	21.1
Non-rheumatic valvular heart disease	50(46–54)	110(102–119)	120.0	150(139.4–161.6)	174.8(162.3–188)	16.6
Other cardiovascular and circulatory diseases	145(115–177)	284(226–349)	96.2	382.5(306–468.8)	470.2(378.4–568.7)	22.9

Note:UIs: Uncertainty Intervals.

The only reduction was observed in the age-standardized prevalence rate of rheumatic heart diseases (−0.3%) in Henan Province from 1990 to 2017. The age-standardized prevalence rate of other subcategories increased significantly from 1990 to 2017, with the largest increase observed in stroke (34.7%), followed by other cardiovascular and circulatory diseases (21.8%), hypertensive heart diseases (20.1%), endocarditis (18.9%) and non-rheumatic valvular heart diseases (17.2%) (Table 1).

Compared with 1990, the subcategory of CVDs with the largest increase in age-standardized prevalence in 2017 for males was stroke, increased by 46.8%, and that for females was cardiomyopathy and myocarditis, increased by 27%.

### CVDs mortality

Deaths caused by CVDs in Henan Province increased by 106.9%, from 181,000 (95% UI: 169–194) in 1990 to 375,000 (95% UI: 324–433) in 2017, among which those of males increased by 125.8% from 88,000 to 199,000, and those of females increased by 89.0% from 93,000 to 176,000 (Table 2). The largest increase was presented in peripheral artery disease (378.3%), followed by ischemic heart diseases (258.7%), atrial fibrillation and flutter (154.2%), aortic aneurysm (92.7%), other cardiovascular and circulatory diseases (83.5%), stroke (71.0%), non-rheumatic valvular heart disease (57.7%), cardiomyopathy and myocarditis (31.4%), and endocarditis (23.8%). A significant decline was observed in rheumatic heart diseases and hypertensive heart diseases, which decreased by 45.0% and 8.1%, respectively (Table 2).

**Table 2.** Deaths and age-standardized mortality rate and percentage change between 1990 and 2017 for genders combined and separated for cardiovascular diseases in Henan

Groups	Deaths, in Thousands (95% UIs)			Age-Standardized Mortality Rate (95% UIs), per 100,000		
	1990	2017	Percentage Change (%)	1990	2017	Percentage Change (%)
Both genders						
Cardiovascular diseases	181(169–194)	375(324–433)	106.9	355(331.6–379.1)	364.1(315.2–417.5)	2.6
Rheumatic heart disease	5(5–6)	3(2–3)	−45.0	8.7(7.8–9.7)	2.6(2.2–3)	−69.9
Ischemic heart disease	45(41–49)	162(140–187)	258.7	90.3(83.1–98)	161.8(140.4–185.5)	79.2
Stroke	108(101–117)	185(159–214)	71.0	206.9(191.3–222.7)	173.2(149.5–198.9)	−16.3
Hypertensive heart disease	19(10–22)	17(13–20)	−8.1	40(22.9–48.2)	17.7(12.7–20.9)	−55.8
Cardiomyopathy and myocarditis	1(1–1)	1(1–1)	31.4	1.2(1–1.5)	1.1(0.9–1.3)	−12.8
Atrial fibrillation and flutter	1(1–1)	3(3–4)	154.2	3.4(2.7–4)	3.6(3.1–4.2)	5.1
Aortic aneurysm	0(0–1)	1(1–1)	92.7	0.8(0.6–1.1)	0.8(0.7–1)	−2.2
Peripheral artery disease	0(0–0)	0(0–0)	378.3	0.1(0–0.1)	0.1(0.1–0.3)	115.4
Endocarditis	0(0–0)	0(0–0)	23.8	0.4(0.4–0.5)	0.3(0.3–0.4)	−20.0
Non-rheumatic valvular heart disease	0(0–1)	1(0–1)	57.7	0.7(0.5–1.1)	0.5(0.4–0.9)	−23.4
Other cardiovascular and circulatory diseases	1(1–2)	2(2–3)	83.5	2.4(1.9–3.1)	2.4(1.9–2.8)	−1.9
Males						
Cardiovascular diseases	88(80–96)	199(162–240)	125.8	392.8(360.1–427.8)	423.9(348.6–506)	7.9
Rheumatic heart disease	2(2–2)	1(1–1)	−39.9	8(7–9)	2.6(2.1–3.1)	−67.8
Ischemic heart disease	24(21–27)	85(69–103)	258.0	105.1(94.6–118.7)	186.3(153.4–223.6)	77.2
Stroke	52(47–58)	101(82–121)	93.0	229.3(208.4–252.5)	206(168.9–245.5)	−10.2
Hypertensive heart disease	8(4–10)	8(6–10)	1.5	41.3(23.3–49.3)	19.5(14.1–23.9)	−52.7
Cardiomyopathy and myocarditis	0(0–1)	1(1–1)	44.1	1.4(1.1–2)	1.4(1.1–1.9)	0.8
Atrial fibrillation and flutter	0(0–0)	1(1–1)	200.4	2.4(1.7–3.1)	2.7(2.2–3.3)	14.1
Aortic aneurysm	0(0–0)	1(1–1)	106.2	1.3(0.9–1.8)	1.3(1–1.7)	1.5
Peripheral artery disease	0(0–0)	0(0–0)	471.6	0.1(0–0.2)	0.2(0.1–0.4)	148.8
Endocarditis	0(0–0)	0(0–0)	25.9	0.5(0.4–0.6)	0.4(0.4–0.5)	−17.7
Non-rheumatic valvular heart disease	0(0–0)	0(0–1)	62.9	0.8(0.6–1.4)	0.6(0.5–1.1)	−21.6
Other cardiovascular and circulatory diseases	1(1–1)	1(1–2)	107.3	2.6(1.9–3.6)	2.9(2.2–3.7)	11.0
Females						
Cardiovascular diseases	93(84–103)	176(144–213)	89.0	325.3(294–358.5)	312(255.6–376.1)	−4.1
Rheumatic heart disease	3(3–3)	2(1–2)	−48.4	9.4(8.2–10.8)	2.7(2.1–3.2)	−71.8
Ischemic heart disease	21(19–24)	77(63–93)	259.5	78.3(68.9–87.7)	139.9(114.7–168.2)	78.7
Stroke	56(50–62)	84(68–103)	50.5	190.3(169–211.6)	145.2(118.2–176.8)	−23.7
Hypertensive heart disease	10(5–13)	9(5–11)	−15.7	38.5(20.6–49.3)	16.1(9.9–20.1)	−58.3
Cardiomyopathy and myocarditis	0(0–0)	0(0–0)	15.4	1.1(0.9–1.3)	0.8(0.6–0.9)	−25.9
Atrial fibrillation and flutter	1(1–1)	2(2–3)	138.2	4(2.9–4.8)	4.2(3.4–5)	4.0
Aortic aneurysm	0(0–0)	0(0–0)	64.4	0.5(0.3–0.7)	0.4(0.3–0.5)	−16.6
Peripheral artery disease	0(0–0)	0(0–0)	294.6	0.1(0–0.1)	0.1(0.1–0.2)	83.2
Endocarditis	0(0–0)	0(0–0)	20.6	0.3(0.3–0.4)	0.2(0.2–0.3)	−25.2
Non-rheumatic valvular heart disease	0(0–0)	0(0–0)	52.3	0.6(0.4–1)	0.4(0.3–0.8)	−26.1
Other cardiovascular and circulatory diseases	1(0–1)	1(1–1)	59.7	2.2(1.6–3)	1.9(1.5–2.4)	−14.4

Note: UIs: Uncertainty Intervals.

**Figure 1.**
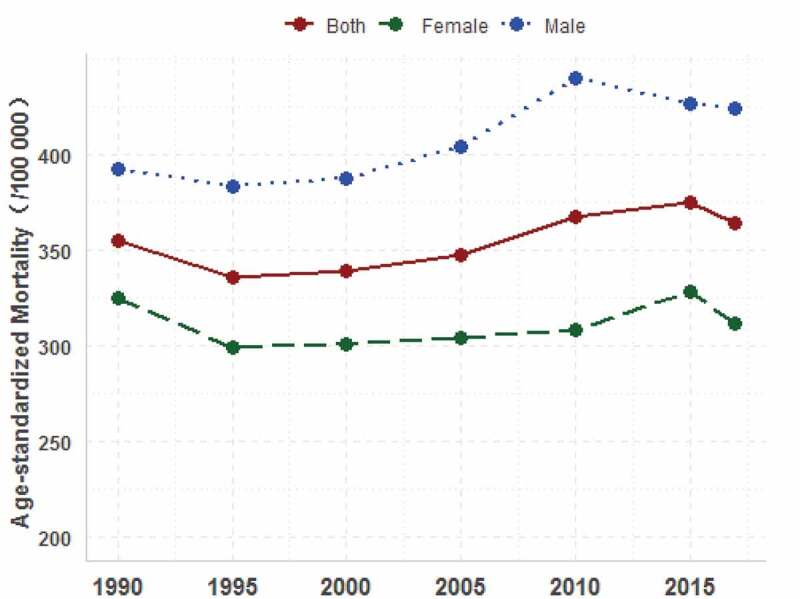
Tendency of cardiovascular diseases age-standardized mortality among residents in Henan, 1990–2017

The age-standardized mortality rate caused by CVDs increased by 2.6% from 355.0 (95% UI: 331.6–379.1) per 100,000 in 1990 to 364.1 (95% UI: 315.2–417.5) per 100,000 in 2017 (Table 2). As shown in Figure 1, the upward trend of age-standardized mortality rate of CVDs from 1990 to 2017 was seen both for males and females in Henan. The age-standardized CVDs mortality rate of males was higher than that of females. There was a significant reduction in the age-standardized mortality rate in 8 of the 11 subcategories, among which rheumatic heart diseases and hypertensive heart diseases decreased by 69.9% and 55.8%, respectively; compared with 1990, peripheral artery diseases, ischemic heart diseases, atrial fibrillation and flutter in 2017 increased significantly by 115.4%, 79.2% and 5.1%, respectively.

**Figure 2.**
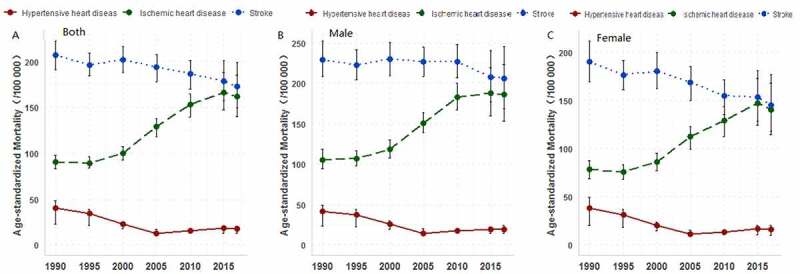
Change trends of age-standardized mortality for main cardiovascular diseases in Henan, 1990–2017. (A) age-standardized mortality for both males and females. (B) age-standardized mortality for for males. (C) age-standardized mortality for females

Stroke and ischemic heart diseases were the main causes of CVDs deaths in Henan Province in 2017 (age-standardized mortality: 173.2 per 100,000, and 161.8 per 100,000, respectively). Stroke and ischemic heart diseases contributed to 92.5% of total CVDs deaths (Table 2). The total CVDs mortality rate for male residents in Henan was higher than that of females, and the mortality rate of ischemic heart diseases, stroke, and hypertensive heart diseases for males were significantly higher than females, while that of rheumatic heart diseases and atrial fibrillation for males were lower than for females. (Table 2). The trend analysis of CVDs subcategories showed that regardless of gender, the obvious upward trend was seen in the age-standardized mortality rate of ischemic heart diseases from 1990 to 2017 in Henan, while a downward trend was seen for stroke and hypertensive heart diseases (Figure 2).

### Burden of CVDs

The number of DALYs due to CVDs increased by 75.9% from 4.2 million person-years (95% UI: 3.9–4.5) in 1990 to 7.3 million person-years (95% UI: 6.4–8.4) in 2017. However, the age-standardized DALYs rate fell by 3.9% from 6 515.0 (95% UI: 6095.1–6958.2) per 100,000 in 1990 to 6257.9 (95% UI: 5486.5–7146.9) per 100,000 in 2017, which was significantly higher than that of the national average (5,217/100,000) in 2017. The number of YLDs was 0.8 (95% UI: 0.6–1.0) million person-years in 2017, which was 119.3% higher compared with 1990. The number of YLLs was 6.6 (95% UI: 5.6–7.6) million person-years in 2017, with an increase of 71.8% since 1990. Age-standardized YLDs rate increased by 27.5% from 523.6 (95% UI: 383.3–672.3) per 100,000 in 1990 to 667.8 (95% UI: 489.1–850.3) per 100,000 in 2017. However, age-standardized YLLs rate decreased by 6.7% from 5991.4 (95% UI: 5586.2–6403.9) per 100,000 in 1990 to 5590.2 (95% UI: 4802.1–6486.5) per 100,000 in 2017. The contribution of YLLs to DALYs decreased from 91.4% in 1990 to 89.2% in 2017 (Table 3, Table 4).

**Table 3.** DALYs, YLDs, YLLs, and percentage change between 1990 and 2017 for genders combined and separated for cardiovascular diseases in Henan

Groups	1990 (per thousands)			2017 (per thousands)			Percentage Change (%)		
	YLDs (95% UIs)	YLLs (95% UIs)	DALYs (95% UIs)	YLDs (95% UIs)	YLLs (95% UIs)	DALYs (95% UIs)	YLDs	YLLs	DALYs
Both genders									
Cardiovascular diseases	361 (265–464)	3813 (3555–4079)	4174 (3893–4461)	792(577–1009)	6551 (5599–7639)	7343 (6407–8406)	119.3	71.8	75.9
Rheumatic heart disease	32 (21–47)	142 (129–156)	174 (156–195)	38(25–56)	54(46–63)	92 (76–111)	18.3	−62.1	−47.2
Ischemic heart disease	37 (25–51)	966 (883–1057)	1003 (922–1097)	72(50–99)	2735 (2335–3201)	2807 (2412–3284)	92.8	183.3	179.9
Stroke	236 (170–303)	2261 (2098–2431)	2496 (2310–2680)	568(407–726)	3348 (2841–3909)	3916 (3408–4472)	140.9	48.1	56.9
Hypertensive heart disease	14 (10–21)	312(174–371)	327 (187–386)	33(23–47)	260(194–309)	293 (225–344)	132.0	−16.8	−10.3
Cardiomyopathy and myocarditis	3 (2–4)	38(31–51)	41(33–54)	4(3–5)	32(27–39)	36(31–44)	47.3	−14.7	−10.8
Atrial fibrillation and flutter	18 (12–25)	15(12–18)	33(26–40)	38(25–53)	35(30–41)	73(60–88)	115.2	128.3	121.3
Aortic aneurysm	0 (0–0)	11(8–15)	11(8–15)	0(0–0)	19(15–24)	19(15–24)		66.3	66.3
Peripheral artery disease	5 (2–9)	0(0–1)	5(3–9)	8(4–14)	2(1–4)	10(5–17)	62.9	329.2	87.2
Endocarditis	0 (0–0)	15(12–20)	15(12–20)	0(0–0)	8(7–10)	8(7–10)	62.2	−46.6	−45.3
Non-rheumatic valvular heart disease	1 (1–2)	8(6–12)	9(7–13)	2(1–3)	10(8–16)	12(10–18)	106.8	26.5	36.2
Other cardiovascular and circulatory diseases	15 (10–22)	45(34–65)	60(47–82)	29(19–42)	48(39–56)	76(63–91)	89.0	6.8	27.5
Males									
Cardiovascular diseases	158 (115–203)	2052(1861–2260)	2210 (2015–2421)	363(263–464)	3862 (3080–4732)	4224 (3452–5092)	129.6	88.2	91.2
Rheumatic heart disease	15 (10–22)	61(53–69)	75(65–86)	16(11–24)	25(20–31)	42(34–51)	11.0	−58.4	−44.8
Ischemic heart disease	17 (11–23)	571(507–650)	587(522–666)	30(21–42)	1632 (1307–2024)	1662 (1340–2054)	83.2	185.9	183.0
Stroke	102 (73–132)	1192(1071–1326)	1294 (1171–1427)	267(191–344)	1973 (1583–2414)	2240 (1834–2677)	160.6	65.5	73.0
Hypertensive heart disease	5 (3–8)	154(85–185)	160(89–189)	12(8–18)	143(101–177)	155 (113–191)	134.2	−7.4	−2.9
Cardiomyopathy and myocarditis	1 (1–2)	22(17–37)	23(19–38)	2(1–3)	22(17–28)	23(19–30)	47.0	−2.3	0.4
Atrial fibrillation and flutter	8 (5–11)	4(3–6)	12(9–16)	17(11–24)	12(9–15)	29(22–36)	116.0	168.8	135.1
Aortic aneurysm	0 (0–0)	8(6–12)	8(6–12)	0(0–0)	14(11–19)	14(11–19)		75.8	75.8
Peripheral artery disease	2 (1–4)	0(0–1)	2(1–4)	3(2–6)	1(1–3)	5(3–8)	70.7	420.9	110.0
Endocarditis	0 (0–0)	9(7–13)	9(7–13)	0(0–0)	5(4–7)	6(4–7)	59.2	−42.4	−41.4
Non-rheumatic valvular heart disease	0 (0–1)	4(3–8)	5(4–8)	1(1–1)	6(4–10)	7(5–11)	119.4	26.4	34.4
Other cardiovascular and circulatory diseases	7(5–11)	25(19–36)	33(26–44)	14(9–20)	29(21–36)	42(33–52)	83.3	14.4	30.1
Females									
Cardiovascular diseases	203(148–260)	1761(1577–1953)	1965 (1777–2172)	430(312–550)	2689(2160–3286)	3119 (2599–3728)	111.4	52.7	58.7
Rheumatic heart disease	18(11–26)	81(71–94)	99(86–113)	22(14–32)	29(22–35)	50(40–62)	24.3	−64.9	−49.0
Ischemic heart disease	21(14–29)	395(347–446)	415(365–467)	42(28–57)	1103(878–1349)	1145 (927–1390)	100.6	179.5	175.6
Stroke	133(96–171)	1069(952–1186)	1202 (1080–1329)	301(216–384)	1375(1100–1692)	1677 (1401–2004)	125.8	28.7	39.5
Hypertensive heart disease	9(6–13)	158(82–203)	167(92–212)	21(15–30)	117(77–150)	138(98–171)	130.8	−26.0	−17.3
Cardiomyopathy and myocarditis	1(1–2)	16(12–21)	17(13–22)	2(1–3)	11(9–13)	13(11–15)	47.5	−32.0	−25.9
Atrial fibrillation and flutter	10(6–14)	11(8–13)	21(16–25)	21(14–29)	23(19–29)	44(36–53)	114.6	112.1	113.3
Aortic aneurysm	0(0–0)	3(2–4)	3(2–4)	0(0–0)	5(4–6)	5(4–6)		42.1	42.1
Peripheral artery disease	3(1–5)	0(0–0)	3(2–5)	5(2–8)	1(1–2)	5(3–9)	57.4	232.1	70.8
Endocarditis	0(0–0)	6(4–8)	6(4–8)	0(0–0)	3(2–3)	3(2–3)	65.6	−53.3	−51.6
Non-rheumatic valvular heart disease	1(0–1)	3(2–4)	4(3–5)	1(1–2)	4(3–6)	5(4–7)	98.2	26.6	38.6
Other cardiovascular and circulatory diseases	8(5–11)	20(12–33)	27(19–41)	15(10–22)	19(15–24)	34(27–42)	94.6	−3.0	24.5

Notes: DALYs: disability-adjusted life years, YLDs: years lived with disability, YLLs: years of life lost, UIs: Uncertainty Intervals.

**Table 4.** Age-standardized rates of DALYs, YLDs and YLLs per 100,000 and percentage change between 1990 and 2017 for genders combined and separated for cardiovascular diseases in Henan

Groups	1990				2017			Percentage Change(%)		
	YLDs (95% UIs)	YLLs (95% UIs)		DALYs (95% UIs)	YLDs (95% UIs)	YLLs (95% UIs)	DALYs (95% UIs)	YLDs (95% UIs)	YLLs (95% UIs)	DALYs (95% UIs)
Both genders										
Cardiovascular diseases	523.6(383.3–672.3)	5991.4(5586.2–6403.9)	6515(6095.1–6958.2)		667.8(489.1–850.3)	5590.2(4802.1–6486.5)	6257.9(5486.5–7146.9)	27.5(27.6–26.5)	−6.7(−14-1.3)	−3.9(−10-2.7)
Rheumatic heart disease	36.6(23.7–53.1)	196.7(178.2–215.9)	233.3(210.2–257.8)		36.8(24–53.9)	46.7(39.9–54.4)	83.5(68.4–101.1)	0.3(1.2–1.6)	−76.2(−77.6–74.8)	−64.2(−67.5–60.8)
Ischemic heart disease	58(39.7–79.8)	1514.3(1391.5–1648.4)	1572.2(1445.1–1711.2)		59.7(41.4–81.9)	2365.8(2024.7–2752.8)	2425.6(2090.2–2821.7)	3.1(4.4–2.7)	56.2(45.5–67)	54.3(44.6–64.9)
Stroke	341.4(245.5–436)	3550.1(3299.2–3814.5)	3891.5(3604.9–4165.5)		472.7(340.1–604.7)	2802.2(2384.9–3254.3)	3274.9(2852.9–3728)	38.5(38.5–38.7)	−21.1(−27.7–14.7)	−15.8(−20.9–10.5)
Hypertensive heart disease	23.8(16.1–33.6)	544.7(306.4–652.9)	568.5(326.2–673.7)		28.6(19.4–40.4)	230.5(170.1–273.2)	259.1(197.9–303.5)	20.1(20.5–20.3)	−57.7(−44.5–58.2)	−54.4(−39.3–54.9)
Cardiomyopathy and myocarditis	3.2(2.2–4.5)	45.6(37.2–61.4)	48.8(40.1–64.5)		3.7(2.5–5.2)	33.5(28.2–41)	37.2(31.8–44.8)	15.2(13.9–14.9)	−26.5(−24.1–33.2)	−23.7(−20.6–30.5)
Atrial fibrillation and flutter	29.9(20.3–41.8)	33.3(26.6–38.8)	63.3(51.6–76.4)		32.7(22–45.4)	35(29.9–40.8)	67.8(55.9–81)	9.4(8.3–8.4)	5(12.6–5.2)	7.1(8.4–6.1)
Aortic aneurysm	0(0–0)	16.8(12.4–21.6)	16.8(12.4–21.6)		0(0–0)	15.7(12.9–19.4)	15.7(12.9–19.4)	-	−6.3(3.9–10)	−6.3(3.9–10)
Peripheral artery disease	8.4(3.9–15.1)	0.9(0.5–1.9)	9.2(4.7–15.9)		6.8(3.1–12.5)	1.9(1.3–3.6)	8.7(4.7–14.7)	−18.1(−19.4–17.7)	112.2(178.1–93.1)	−5.8(0.6–7.5)
Endocarditis	0.2(0.2–0.3)	17.8(14–22.9)	18(14.2–23.1)		0.3(0.2–0.4)	7.6(6.5–8.9)	7.9(6.8–9.2)	18.7(20.1–17.2)	−57.2(−54–61.2)	−56.2(−52.5–60.1)
Non-rheumatic valvular heart disease	2(1.2–3.1)	11.5(9.2–17.9)	13.5(11.1–20)		2(1.2–3.1)	8.4(6.8–13.9)	10.5(8.5–16.1)	1(0–0.4)	−26.5(−26.2–22.3)	−22.4(−23.4–19.4)
Other cardiovascular and circulatory diseases	20.1(13.1–29)	59.8(46.8–85.1)	79.9(63.9–106.2)		24.4(16–35.7)	42.8(35–49.9)	67.2(55.2–80.1)	21.7(21.8–22.8)	−28.5(−25.3–41.3)	−15.9(−13.6–24.5)
Males										
Cardiovascular diseases	475.8(348.2–612.7)	6808.1(6190.9–7451.9)	7283.9(6656.3–7936.2)		645.1(471.4–823.3)	6965.8(5603.7–8480.9)	7610.9(6255.4–9109.5)	35.6(35.4–34.4)	2.3(−9.5–13.8)	4.5(−6-14.8)
Rheumatic heart disease	32.4(21–47.3)	174(152.3–196.3)	206.4(181.1–233.5)		32(20.8–46.9)	46.7(37.7–57)	78.7(64.3–97.1)	−1.2(−1.2–0.8)	−73.1(−75.2–71)	−61.9(−64.5–58.4)
Ischemic heart disease	54.2(37.1–75.3)	1853.7(1650.2–2095.8)	1907.9(1706.9–2157.1)		53.5(37.2–73.8)	2979.8(2405.3–3670.5)	3033.3(2455.9–3723.2)	−1.2(0.2–2)	60.7(45.8–75.1)	59(43.9–72.6)
Stroke	308.4(220.4–393.2)	3986.8(3606.1–4409.6)	4295.3(3900.7–4729.5)		469.8(334.8–604.8)	3495(2827.1–4240.5)	3964.8(3265.9–4713)	52.3(52–53.8)	−12.3(−21.6–3.8)	−7.7(−16.3–0.3)
Hypertensive heart disease	17.8(11.7–25.8)	589.9(326.9–704.7)	607.8(343.4–719.6)		21.7(14.5–31.5)	271.2(193.3–335.1)	292.9(214.1–357.8)	21.8(24–22.4)	−54(−40.9–52.4)	−51.8(−37.7–50.3)
Cardiomyopathy and myocarditis	3.1(2.1–4.5)	51.6(40.9–81.5)	54.7(44.1–84.3)		3.6(2.5–5.2)	44.1(35–56.8)	47.7(38.5–60.9)	16(16.5–16)	−14.6(−14.3–30.2)	−12.9(−12.7–27.8)
Atrial fibrillation and flutter	29.9(19.7–41.9)	23.1(16.6–30.7)	52.9(41.1–67)		31.8(21.1–45)	26.8(21.8–33.2)	58.6(47–71.7)	6.5(7.1–7.5)	16.1(31.5–8.1)	10.6(14.5–7.1)
Aortic aneurysm	0(0–0)	25.4(17.7–34.8)	25.4(17.7–34.8)		0(0–0)	25.3(19.5–32.9)	25.3(19.5–32.9)	-	−0.4(10.6–5.5)	−0.4(10.6–5.5)
Peripheral artery disease	7.7(3.5–13.9)	1(0.4–2.8)	8.7(4.4–15.2)		6.3(2.9–11.7)	2.5(1.5–5.7)	8.8(4.9–15)	−18.2(−18.2–15.6)	149.6(283.5–99)	0.7(12.5–1.5)
Endocarditis	0.3(0.2–0.4)	21.5(16.1–28.8)	21.7(16.4–29.1)		0.3(0.2–0.4)	10.4(8.3–12.8)	10.7(8.6–13.1)	17.7(17.7–16.2)	−51.8(−48.5–55.6)	−50.9(−47.2–55)
Non-rheumatic valvular heart disease	1.9(1.1–3)	13.9(10.5–24.2)	15.8(12.2–26)		2(1.1–3)	10.5(7.9–18.8)	12.5(9.6–20.6)	2.6(2.2–0.1)	−24.4(−25.2–22.7)	−21.1(−21.2–20.6)
Other cardiovascular and circulatory diseases	20.1(13–29.3)	67.2(51.7–93.7)	87.3(69.4–114)		24(15.6–35)	53.5(39.8–66.6)	77.6(61.5–94.5)	19.6(20–19.4)	−20.3(−22.9–28.9)	−11.1(−11.4–17.1)
Females										
Cardiovascular diseases	565.6(411.7–721.9)	5259.8(4727.3–5820.9)	5825.3(5272.9–6440.1)		688.6(502.2–881.1)	4345(3510.1–5300.6)	5033.6(4198.4–6008)	21.8(22–22.1)	−17.4(−25.7–8.9)	−13.6(−20.4–6.7)
Rheumatic heart disease	41.1(26.5–59.2)	220.4(191.8–253.9)	261.5(227.4–299.6)		41.5(26.8–60.6)	46.6(36.6–57.1)	88(69.2–108.6)	1(1.4–2.3)	−78.9(−80.9–77.5)	−66.3(−69.6–63.8)
Ischemic heart disease	61.1(42–84.6)	1200.1(1057.9–1346.5)	1261.2(1117.9–1411.8)		65.1(44.4–89.4)	1807.3(1446.2–2204.5)	1872.4(1525.5–2265.6)	6.5(5.8–5.7)	50.6(36.7–63.7)	48.5(36.5–60.5)
Stroke	370.6(267.5–475)	3171.5(2829.2–3518.1)	3542(3189.7–3916.9)		476(342.8–608.4)	2179.7(1745.5–2678.4)	2655.7(2221.4–3172.2)	28.5(28.2–28.1)	−31.3(−38.3–23.9)	−25(−30.4–19)
Hypertensive heart disease	28.2(19.2–39.5)	502.7(264.1–638.2)	530.9(292.4–668.7)		34.2(23.5–47.9)	193.8(125.6–247.2)	228(160.9–281.5)	21.5(22.6–21.1)	−61.5(−52.4–61.3)	−57.1(−45–57.9)
Cardiomyopathy and myocarditis	3.2(2.2–4.6)	39.3(29.8–50.9)	42.5(33–54.6)		3.7(2.4–5.4)	23.3(19.1–28.4)	27.1(22.7–32.5)	15.2(12.3–16.1)	−40.6(−35.8–44.3)	−36.3(−31.1–40.5)
Atrial fibrillation and flutter	30.1(20.2–42.3)	39.8(29.6–47.7)	70(55.4–84.7)		33.7(22.5–46.7)	41(33.2–50.1)	74.7(60.7–89.8)	11.8(11.6–10.5)	3(12–4.8)	6.8(9.7–6.1)
Aortic aneurysm	0(0–0)	9.3(6.3–11.8)	9.3(6.3–11.8)		0(0–0)	7.2(5.6–9)	7.2(5.6–9)	-	−22(−10.9–23.5)	−22(−10.9–23.5)
Peripheral artery disease	8.8(4.1–16)	0.8(0.3–1.5)	9.6(4.8–16.7)		7.3(3.3–13.2)	1.3(0.9–2.6)	8.6(4.6–14.7)	−17.5(−19.6–17.9)	68.1(179.9–73)	−10.5(−5.4–12.2)
Endocarditis	0.2(0.1–0.3)	14.1(10.1–19.5)	14.3(10.3–19.7)		0.3(0.2–0.4)	5(4–6.2)	5.3(4.3–6.5)	20.7(21.9–21.8)	−64.2(−60.3–68.3)	−62.9(−58.5–67.2)
Non-rheumatic valvular heart disease	2.1(1.2–3.3)	9.2(7.3–13.8)	11.2(9.1–15.9)		2.1(1.2–3.3)	6.5(4.9–10.7)	8.6(6.7–12.7)	0.4(0–0.4)	−29(−31.9–22.3)	−23.6(−27–20.5)
Other cardiovascular and circulatory diseases	20.2(13.1–29.3)	52.6(33.4–85.3)	72.7(51.9–106.7)		24.8(16.3–36.2)	33.2(26.2–41)	58(46.1–70.8)	22.8(24.3–23.2)	−36.9(−21.8–52)	−20.3(−11.2–33.6)

Notes: DALYs: disability-adjusted life years, YLDs: years lived with disability, YLLs: years of life lost, UIs: Uncertainty Intervals.

Stroke and ischemic heart diseases were the predominant contributors of CVDs in Henan Province in 2017 (age-standardized DALYs: 3274.9 (95% UI: 2852.9–3728) per 100,000, and 2425.6 (95% UI: 2090.2–2821.7) per 100,000, respectively). In 2017, stroke and ischemic heart diseases caused 52.3% (95% UI: 52.0–52.2) and 38.8% (95% UI: 38.1–39.5) of the total CVDs DALYs, respectively, accounting for 91.1% (95% UI: 90.1–91.6) in total (Table 3, Table 4). The age-standardized YLDs rate in females was significantly higher than that in males, but the age-standardized YLLs rate was higher in males than that in females for most subcategories except for atrial fibrillation and flutter (Table 4).

### Risk factors of CVDs

**Figure 3.**
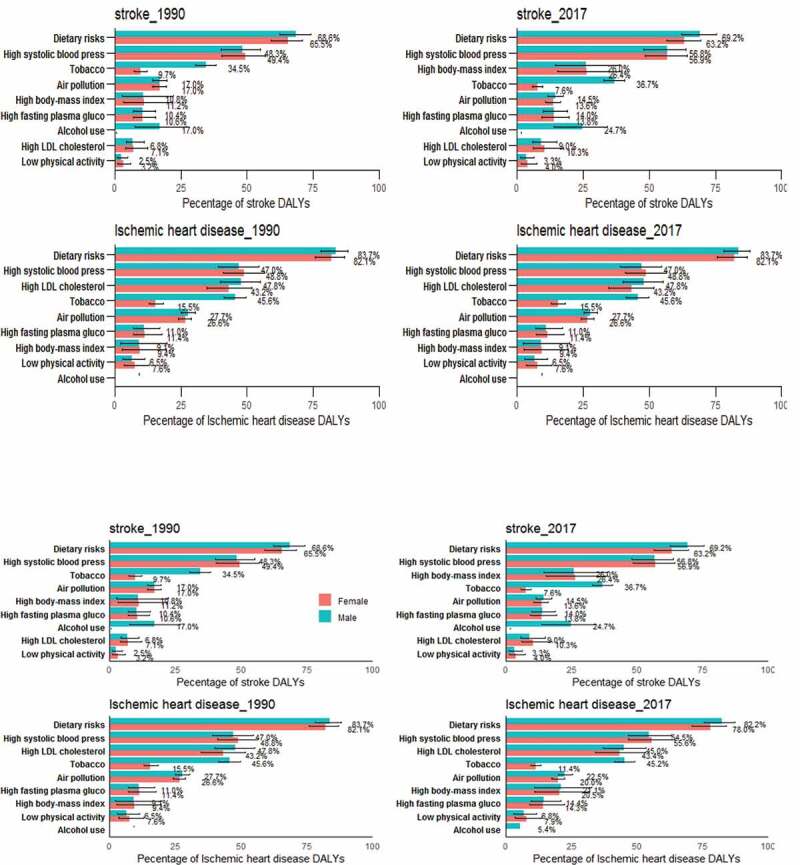
Percentage attribution of major risk factors of ischemic heart disease and stroke DALYs in Henan by gender in 1990 and 2017

The most common risk factors of CVDs in Henan Province were dietary risks, high systolic blood pressure, and tobacco use, with attributable fraction of 69.4% (95% UI: 64.2%-74.1%), 56.7% (95% UI: 50.3%-62.0%), and 25.2% (95% UI: 22.3%-28.1%), respectively in 2017. The leading risk factors of DALYs attributable to stroke in 2017 were dietary risks, high systolic blood pressure, high body mass index (BMI), and tobacco use. For ischemic heart diseases, risk factors were dietary risks which mainly contain high intake of sodium, alcohol use, low intake of fruits, whole grains, vegetables, nuts and seed, high systolic blood pressure, high low-density lipoprotein cholesterol (LDL-C), and tobacco use (Figure 3). The proportion of DALYs attributable to dietary risks, high LDL-C, air pollution and tobacco use for ischemic heart diseases was much greater than that for stroke. The proportional contribution of tobacco use to ischemic heart diseases and stroke DALYs was larger for males than for females. The proportion of stroke DALYs attributable to alcohol use was much greater than that of ischemic heart diseases.

## Discussion

Henan Province has entered a new stage of rapid social and economic development and is faced with a huge burden of noncommunicable diseases. The findings in this study provide a systematic understanding of CVDs burden and the major risk factors in Henan Province from 1990 to 2017 using all the data available in the GBD 2017..

### Characteristics of the overall burden

The standardized DALYs rate caused by CVDs in Henan Province showed a downward trend from 1990 to 2017, which was consistent with that in the whole country [2]. However, the absolute burden caused by cardiovascular diseases in Henan has shown an obvious upward trend, and the absolute number of all-age deaths and YLDs caused by CVDs per 100,000 in Henan has more than doubled since 1990. The burden of CVDs on premature deaths was significantly greater than that on disability during the study period, but the burden on disability increased faster than that on premature deaths. Rapid and sustained economic development, health improvement, and population aging were likely to have contributed to these changes. The sixth population census of China in 2000 illustrated that the proportion of population aged 60 and above in Henan was 12.7% and for aged 65 and over was 8.4% [21]. The rapid increase in disability burden caused by CVDs can be partly explained by population aging and improvements in life expectancy in Henan Province.

This study found that the CVDs burden of male residents in Henan Province was greater than that of females, which was consistent with other reports [2]. The main manifestation was that the number of YLLs of males was significantly higher than that for females, and the age-standardized YLLs rate of males showed an increasing trend compared with 1990, while that of females showed a downward trend. This trend can be partly explained by different exposure levels of risk factors such as smoking, excessive drinking, and mental stress in different genders [22]. Previous studies have shown that smoking, dyslipidemia, and hypertension were the top three risk factors of stroke for males aged 40 and above, while for females were dyslipidemia, hypertension, and obvious overweight or obesity [23]. This study showed that the top three risk factors of stroke for males in Henan were dietary risks, high systolic blood pressure and tobacco use, while for females were dietary risks, high systolic blood pressure and high BMI [23]. It is suggested that healthy diet and the prevention and treatment of hypertension be determined as CVDs prevention and control strategies in Henan. Additionally, gender differences should be considered in the CVDs prevention and control in Henan. More attention should be paid to smoking control in males and increasing physical activity and maintaining a healthy weight in females.

### Characteristics of subcategories

It was found that stroke and ischemic heart diseases were the top two CVDs in Henan, accounting for 91.6% of the total CVDs burden, which was consistent with previous national and other provincial studies [5,9]. Compared with other similar developing countries, the characteristics of CVDs burden of Henan Province in China are not exactly the same. Previous studies found that the total death and disease burden caused by CVDs in every state of India had been significantly increased since 1990, which was similar to that of Henan Province in China, but the leading cause of disease burden in India in 2016 was ischemic heart diseases, and stroke was the fifth leading cause [24,25]. This can be partly explained by the differences in health levels, dietary habits and lifestyles between India and China.

The burden of stroke ranked first among all CVDs in Henan Province and increased obviously, especially YLDs rate. On the one hand, it is closely related to the rapid social and economic development with the improvement in medical treatment and the substantial increase in residents’ life expectancy in the past decades in China. On the other hand, the measures have been taken to raise public awareness of CVDs, strengthen early detection, early intervention and management of patients, reduce stroke mortality, improve survival rates, and increase the burden of disability from stroke, including strengthening the construction of stroke centers in secondary and tertiary hospitals in Henan, constantly improving the capabilities of stroke treatment, and carrying out ‘China National Lifestyle Action’ and the China national stroke screening and intervention program [26–28]. The study showed that the burden of both premature death and disability caused by CVDs in Henan were severe. As a result, more attention should be paid to the prevention and control of stroke and ischemic heart diseases to reduce the burden.

### Risk factors

It was found that unhealthy diet, high systolic blood pressure, and tobacco use were major risk factors of stroke and ischemic heart diseases. The main dietary risk factors of stroke were high intake of sodium, low intake of fruits, whole grains, vegetables, and alcohol use. The main dietary risk factors of ischemic heart diseases included high intake of sodium, low intake of nuts and seed, whole grains, seafood omega, fruits, fiber, vegetables, polyunsaturated fatty acids, and legumes. A prospective cohort study has shown that the prevalence of hypertension among population aged 35 to 74 in China was 32.5%, and uncontrolled hypertension was associated with relative risks of CVDs mortality [28]. Our findings indicated that the stroke and ischemic heart diseases burden attributable to tobacco use among males was significantly greater than that among females (36.7% and 45.2% of stroke and ischemic heart diseases were attributed to tobacco use among males and 7.6% and 11.4% among females in 2017). This can be explained by the difference in tobacco use between the genders. According to the ‘2010 Global Adult Tobacco Survey’, the smoking rate in China was 52.9% among males, which was much higher than 2.4% among females [22]. In 2015, the smoking rate of males aged 15 to 69 in Henan Province was 44.49%, while that of females was 1.26% [29]. The smoking rate of males was 35 times higher than that of females [29]. This suggests that the task of tobacco control in Henan Province is exceptionally arduous.

High BMI mainly increases the risk of stroke, while high LDL-C has a greater impact on the risk of ischemic heart diseases. Several studies have shown that BMI is an independent risk factor of stroke [30,31]. BMI in adolescence could increase the risk of stroke in adulthood, which was associated with both ischemic stroke events (HR per SD increase 1.19; 95% CI: 1.11–1.28) and intracerebral hemorrhage events (HR per SD increase 1.29; 95% CI: 1.15–1.46). It is proposed that greater BMI during puberty contributes to an increased risk of adult stroke, at least partly intermediated by increased blood pressure [30].

A previous study reported that the overall level of health literacy was 3% among residents of Henan Province in 2014 [32]. A survey of behavioral risk factors in six provinces of China, including Henan Province, showed that 57.6% of people exercised less than three times a week, 21.3% did not have a regular diet, 58.7% did not pay attention to diet control, and 15.7% and 7.8% preferred salty and greasy diet [33]. It is important to note that the cumulative effect of these risk factors is less than the simple sum of their contribution, as the risk factors overlap.

## Limitations

GBD used unified and standardized methods to make the estimates comparable across provinces and time periods. However, as the data were derived from GBD 2017, this study shared many limitations of GBD methodology as described previously [6,7,9]. First, the data sources used to estimate provincial burden of CVDs failed to include provincial cardiovascular event surveillance data, which might impact the accuracy of the data and underestimate the burden of CVDs. Second, although China has carried out a pilot project of national cardiovascular disease surveillance since 2014, data on the non-fatal consequences of CVDs were still seriously insufficient. The estimation of the incidence and prevalence of CVDs subcategories highly depended on the regional patterns and predictors in the models [2]. Third, in aggregating the PAFs of multiple behavioral and metabolic risk factors, they were assuming as independent risk factors without mediating effect, which led to the combined effects of multiple risk factors not being taken into account [6].

## Conclusion

This study suggests that the prevention and control of CVDs in Henan Province should focus on stroke and ischemic heart diseases, and comprehensive prevention and control strategies. Health policy should pay more attention to balancing diet, quitting smoking, controlling blood pressure and blood lipids, and maintaining a healthy weight. The management of blood lipids should be strengthened to prevent and control ischemic heart diseases and ischemic stroke. In particular, the propaganda and intervention on drinking less or no alcohol should be strengthened to prevent and control high LDL-C and hemorrhagic stroke. Dietary intervention should focus on reducing salt and increasing the intake of nuts, fruits and whole grains.

## Paper context

Nationally cardiovascular diseases are the leading causes of premature death and disability in China. It is also important to gain evidence of the burden at the provincial level so that prevention and control strategies can be put in place. This study of the cardiovascular disease burden and associated risk factors in Henan province, recommends that public health policies should target obesity, smoking and alcohol consumption in order to reduce the burden from cardiovascular disease.
